# Dissecting genotype-specific effects of disease-associated genetic variants

**DOI:** 10.1016/j.isci.2026.116143

**Published:** 2026-06-01

**Authors:** Sophie L. Farrow, Sreemol Gokuladhas, Izlem Su Akan, Denis Nyaga, Antony A. Cooper, Ralph Stefan Grand, Justin M. O’Sullivan

**Affiliations:** 1 Liggins Institute, The University of Auckland, Auckland 1023, New Zealand; 2 Oxford Parkinson’s Disease Centre and Department of Physiology, Anatomy and Genetics, University of Oxford, Oxford OX1 3QU, UK; 3 Kavli Institute for Neuroscience Discovery, Dorothy Crowfoot Hodgkin Building, University of Oxford, South Parks Road, Oxford OX1 3QU, UK; 4 Centre for Molecular Biology of Heidelberg University (ZMBH), University of Heidelberg, 69120 Heidelberg, Germany; 5 Australian Parkinsons Mission, Garvan Institute of Medical Research, Sydney, NSW, Australia; 6 St Vincent’s Clinical School, University of New South Wales, Sydney, NSW, Australia; 7 Singapore Institute for Clinical Sciences, Agency for Science Technology and Research, Singapore, Singapore; 8 MRC Lifecourse Epidemiology Unit, University of Southampton, Southampton, UK

**Keywords:** Human genetics, Non-infectious disease, Clinical neuroscience

## Abstract

Non-coding variants associated with complex disease can shape gene regulatory networks across multiple genomic loci and cellular contexts. Here, we investigated the functional impact of the Parkinson-disease-associated variant rs11610045 using an isogenic induced pluripotent stem cell model. Using CRISPR-Cas9 editing and reversal, we generated matched clones carrying either the A|A or G|G genotype, enabling controlled comparison of allele-specific effects. We identified widespread genotype-dependent regulation of distal genes, including *THBS1* and *PDGFB*. Affinity purification followed by mass spectrometry revealed differential binding of regulatory proteins to the G|G allele, including the transcription factor TCF7L1. Differentiation into cortical neurons demonstrated context-dependent effects, with 24 genes differentially expressed and *PAX5* consistently altered across developmental stages. Together, these findings link a non-coding disease-associated variant to coordinated changes in gene expression and protein binding, support *trans-*acting mechanisms underlying regulatory variation, and provide a generalizable framework for dissecting disease-associated loci in human cellular models.

## Highlights

• Rs11610045 drives allele-specific regulation of distal target genes
• Affinity purification-MS identifies allele-specific binding of TCF7L1 at rs11610045
• Variant-dependent methylation changes associate with altered gene expression
• Neuronal differentiation reveals context-dependent gene expression changes

## Graphical abstract

**Figure:**
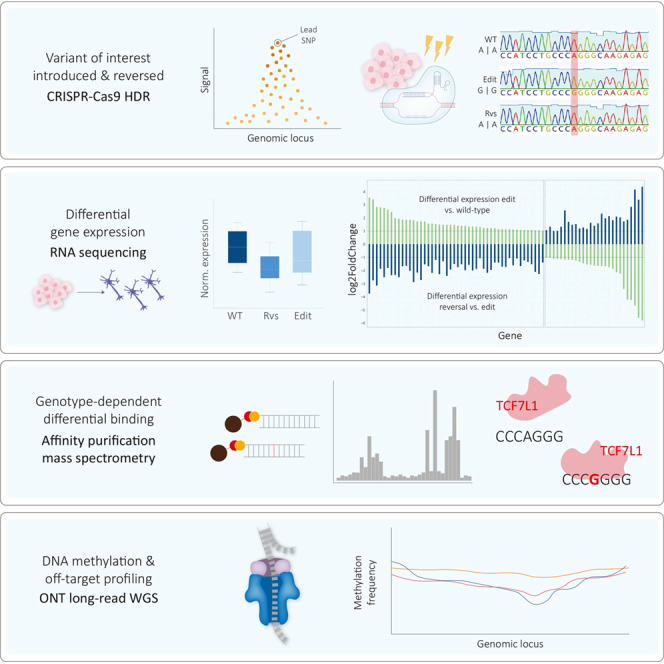

## Introduction

Genome-wide association studies (GWASs) have been instrumental for identifying genetic regulatory variants that contribute to disease risk. However, understanding *how* or *why* these variants increase the risk of complex diseases, such as Parkinson disease (PD), remains a significant challenge.^1^^,^^2^ This difficulty is largely driven by the fact that >90% of all GWAS variants lie in non-coding regions of the genome.^3^

Many tools have been developed for analyzing non-coding variants, including *in silico* (i.e., variant effect prediction tools^4^), *in vitro*, and *in vivo* tools (i.e., massively parallel reporter assay^5^). One such tool that has expanded the scalability of functional studies is CRISPR-based screens. However, these have typically focused on gene knockdown/knockout, using *a priori* assumptions or *in silico* predictions for nominating potential target genes for risk variants.^6^ While there have been several high-throughput studies using CRISPR-based gene knockout screens to elucidate the function of GWAS loci, studies looking directly at the impact of non-coding variants at single-base resolution have been limited.^7^ Nonetheless, the beeSTING-seq method developed by John Morris and colleagues addresses this problem to some extent by integrating cytosine base-editing technology with 10× single-cell RNA sequencing (scRNA-seq).^8^ Despite progress, the beeSTING-seq method is limited to C-T base changes, and CRISPR-based gene knockout screens do not readily enable the investigation of the functional consequences of genomic variants located within non-coding regions or those with unknown significance. Beyond these tools, CRISPR technologies can also be used to introduce single-base changes and enable direct comparisons between isogenic pairs of cell lines that differ only at the regulatory region of interest.^9^ Leveraging these technologies within induced pluripotent stem cells (iPSCs) provides a powerful approach to explore the impact and general molecular underpinnings of risk variants.

Changes to genotypes accumulate throughout the typical life cycle of a cell. However, these changes increase during periods of significant cellular stress, such as those experienced during nucleofection,^10^^,^^11^ which is frequently used in the CRISPR editing process. Long-read genomic sequencing enables the accurate identification of sequence changes across the genome with greater coverage of complex regions, including within non-coding regions.^12^ In addition, the use of the Oxford Nanopore system enables the added benefit of characterization of 5-methylcytosine (5 mC) within the genome, at single-base resolution.^13^ As such, it provides an excellent tool for the identification of accumulated changes within isogenic clones and the changes in 5 mC that are associated with gene expression.

rs11610045 is an intergenic PD-associated SNP located on chr12q24.33, with a minor allele frequency in European populations of 0.498. *FBRSL1* is the nearest gene to rs11610045. However, the SNP lies within a complicated locus that has been reported to have several candidate target genes (including *FBRSL1*, *ULK1*, and *POLE*^14^). To assign genotype-specific function to rs11610045, we utilized CRISPR-Cas9 editing to introduce the alternate genotype for rs11610045 into the KOLF2.1J iPSC line and generate isogenic pairs of cells that were either homozygous wild type (WT) or alternate for this base pair. KOLF2.1J is an iPSC line that has been developed as a common, well-performing, and Mendelian-corrected (correction of mutation in one copy of *ARID2*) reference human cell line for the purpose of large-scale collaborative science.^15^

**Figure 1:**
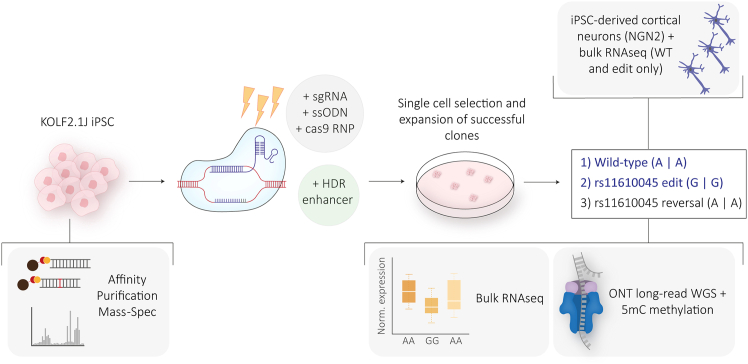
Overview of experimental workflow Affinity purification MS were completed on the undifferentiated KOLF2.1J WT cell line. Bulk RNA-seq and ONT long-read WGS + 5 mC methylation analysis was completed on undifferentiated KOLF2.1J clones containing the following rs11610045 genotypes: A|A (WT), G|G (edited), and A|A (reversed). WT and edited (but not reverse edited) clones were differentiated to iPSC-derived cortical neurons and bulk RNA-seq completed. iPSC, induced pluripotent stem cell; sgRNA, single-guide ribonucleic acid; ssODN, single-strand oligodeoxynucleotide; RNP, ribonucleoprotein; HDR enhancer, homology-directed repair enhancer; ONT, Oxford Nanopore Technologies; WGS, whole-genome sequencing.

Here, we characterized the effects of the rs11610045 genotype in KOLF2.1J iPSCs by generating isogenic pairs through CRISPR-Cas9-mediated introduction and reversal of the SNP. We then integrated a number of molecular approaches: RNA-seq to profile variant-driven differentially expressed genes; long-read whole-genome sequencing to assess mutational load and methylation status; and affinity purification-mass spectrometry (AP-MS) to examine genotype-dependent protein interactions (Figure 1). RNA-seq was also completed in the WT and edited (not reverted) iPSC-cortical neurons. Using this integrative methodology, we identified rs11610045-genotype-specific effects on both nearby and distal target genes, observed in both the induced pluripotent cell state and following differentiation into cortical neurons. Our findings reaffirm previous notions that one SNP can impact the activity of multiple genes,^16^^,^^17^ through both *cis-* and *trans-* mechanisms acting on gene expression and through pathway and protein networks. We also highlight potential candidate genes for future follow-up studies with relevance to PD. This study demonstrates a pipeline to delineate genotype-specific impacts in an iPSC model.

## Results

We focused on PD-SNPs in our experiments following earlier research that identified spatially constrained eQTLs for a subset of PD-SNPs.^16^^,^^17^ We used *in silico* tools^18^ and selection features (i.e., distance to PAM site) to refine our choice to rs11610045. The KOLF2.1J cell line has an A|A genotype (WT) at rs11610045. To introduce the PD-SNP (G|G) into the KOLF2.1J cells, synthetic guide RNAs (sgRNAs) were designed to target rs11610045 and trialed using an *in vitro* CRISPR/Cas9 assay to determine their editing efficiency (Figure S1A). The most efficient sgRNA was used to perform CRISPR base editing with a single-stranded oligodeoxynucleotide (ssODN) repair template that included a G at the rs11610045 position (see methods; Table S1) in the KOLF2.1J cell line. Modified clones, with the rs11610045 G|G (edited) genotype, were isolated by low-density plating and picking. The successful introduction of the rs11610045 G|G genotype was confirmed by Sanger sequencing of the target genomic region in individual clones (Figure S1B).

Table S1. Variant details and CRISPR design details

**Figure 2:**
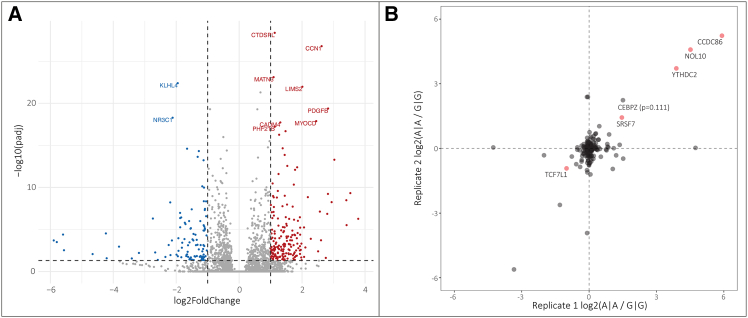
CRISPR-Cas9 editing of the rs11610045 genotype confirms target genes (A) Volcano plot highlighting the 10 genes whose expression is most significantly associated with rs11610045 genotype (see Table S2 for exact *p* values and log2FC values). (B) Scatterplot highlighting proteins with differential binding at the A|A vs. G|G genotype, identified through affinity purification followed by mass spectrometry.

Following the successful generation of rs11610045 G|G genotype containing isogenic cell lines, we used transcriptomics to determine the impact of the rs11610045 genotype compared to the isogenic A|A genotype containing KOLF2.1J cell line. We collected RNA from four edited clones (A4, A5, A8, and A12) and sequenced the polyA-enriched mRNA. To exclude the effects resulting from the process of generating the edited clones (e.g., nucleofection), we exposed the WT iPSC line (rs11610045 A|A genotype) to the same CRISPR/Cas9 editing pipeline, excluding the sgRNA and ssODN, isolated and performed RNA-seq on six technical replicates (six single colonies selected following nucleofection and single-cell plating). Differentially expressed genes (DEGs) were identified by comparing the edited clones (G|G genotype, *n* = 4) with the WT (A|A genotype) nucleofected control (*n* = 6) and another 18 PD-SNP clones (six variants) generated and used as further controls (Table S1 and Figure S1C; not described in this manuscript). This identified 288 DEGs (log2FC > 1; adj. *p* value <0.05; for more precise *p* values see supplementary data) as responding to the rs11610045 genotype (Figure 2 and Table S2).

Table S2. rs11610045 DEGs compared to all other targets (log2FC > 0; *p* > 0.05)

### Long-read whole-genome sequencing identifies mutational load

**Figure 3:**
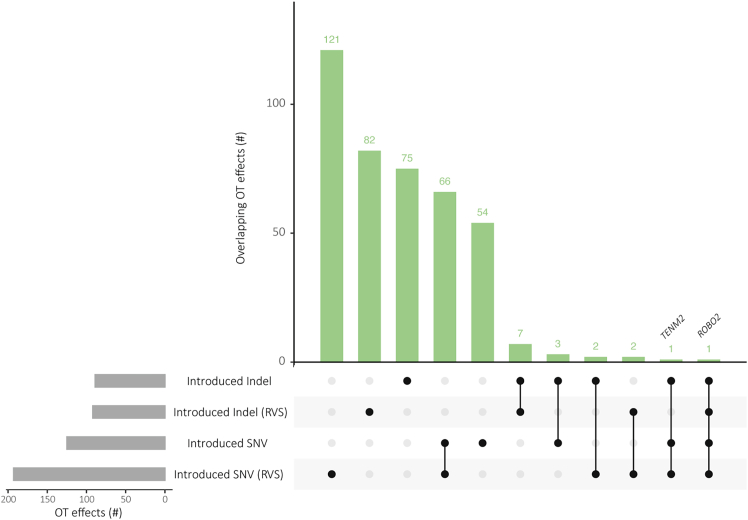
Accumulation of unintended SNVs and indels in edited and reverse-edited lines Intersection between introduced SNVs and indels in the edited rs11610045 line and reverse edited (back to WT) line. Intersection is based on gene location of the SNV or indel.

To identify the potential effects of mutations that randomly accumulate during clonal selection, cell growth, and nucleofection (mutational load, ML), we conducted ONT long-read whole-genome sequencing (WGS) (clone A5). No direct “off-target” modifications resulting from the CRISPR Cas9 sgRNA itself (predicted by using the Cas-OFFinder software^19^) were detected, indicating the sgRNA selection process was effective. Consistent with previous reports,^20^ the in-depth ML analysis revealed multiple unintended “genetic variations” (SNVs and small insertions and deletions [indels]) in addition to our introduced PD-SNP (Figure 3 and Table S3). This unintended genetic variation would have gone unreported if using standard methods (e.g., *in silico* prediction followed by PCR amplification and Sanger sequencing or short-read WGS).

Table S3. rs11610045 introduced variants (identified through ONT long-read sequencing)

To explore whether the accumulated variants impacted gene expression, we compared the overlap between the variants and genes that were differentially expressed in the rs11610045 cell line (see methods). We identified several instances where gene expression changes are likely explained by accumulation of unintended variants, as opposed to the target variant (Figure 3 and Table S4). Following removal of DEGs likely due to ML effects, the number of DEGs was reduced to 283 (Figures 2A, 3, and S2; Tables S2–S4). This analysis highlights the importance of sequencing the whole genome to identify accumulated variants during CRISPR-Cas9 editing procedures, as opposed to more traditional *in silico* prediction and Sanger sequencing methods.

Table S4. DEGs that are likely targets of introduced variation (expression reverts to WT when edit is reversed)

### CRISPR reversal of rs11610045 confirms the SNP-specific regulation of distal gene targets

**Figure 4:**
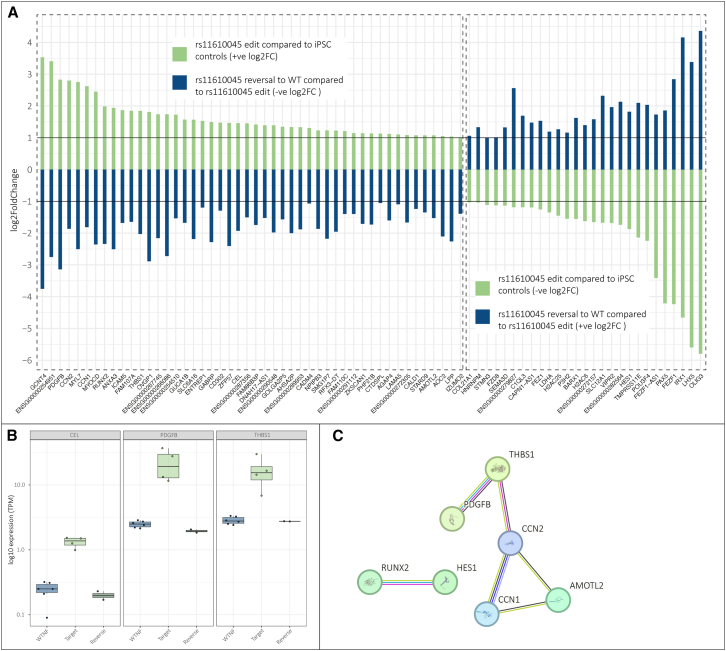
Restoring the rs11610045 G|G genotype to A|A also restores gene expression for a subset of genes (A) CRISPR-Cas9 editing of the rs11610045 G|G genotype to A|A restored the expression of 75 genes to WT levels. Green bars represent the log2FC when comparing the edited rs11610045 G|G genotype with that of the A|A genotype (WT) KOLF2.1J cells. Blue bars represent the log2FC when comparing gene expression in the rs11610045 A|A (reversed) with that of the rs11610045 G|G genotype (edit) clones. In all instances, log2FC > 1 and Bonferroni adj. *p* value <0.05. (B) Expression of *CEL*, *PDGFB*, and *THBS2* are rs11610045 genotype dependent and have been linked to PD. Boxplots show the median (center line), interquartile range (box), and whiskers extending to 1.5× the interquartile range. Individual data points are overlaid. (C) PDGFB interacts with THBS1 in a protein-protein interaction network (StringDB, high confidence interaction score ≥0.700).

To rigorously test the specific impact of rs11610045 on gene activity, we reasoned that if the genotype at rs11610045 were responsible for the observed changes in gene expression, then reverting the genotype in the modified cell line to (G|G) should reverse those changes. This approach will both clarify the impact of ML mutations on gene expression and enable us to isolate the changes induced by specific SNPs. Therefore, we edited the rs11610045 G|G genotype in clones A4 and A5 to reverse it back to the wild-type genotype (i.e., A|A). RNA-seq from two of the reversed A|A genotype KOLF2.1J clones confirmed that 75 of the 283 DEGs (log2FC > 1; adj. *p* value <0.05) returned to expression levels similar to those seen in the original KOLF2.1J A|A genotype WT (Figures 4A and 4B and Table S5). This is consistent with a genotype-specific impact of the rs11610045 SNP on the regulation of these genes. Long-read genome sequencing of the reversed clone A5 identified that no off-target CRISPR edits or unintended variants were present within the DEGs.

Table S5. rs11610045 DEGs with reversal

Genes whose expression was dependent on rs11610045 genotype included *CEL* (log2FC = 1.46), previously associated with early-onset PD in a Finnish GWA study^21^; *THBS1* (log2FC = 1.84), which has suggested interactions with multiple PD-related genes^22^; and *PDGFB* (log2FC = 2.83), which is included in the genomics England PD gene panel (https://panelapp.genomicsengland.co.uk/panels/39/) as a high evidence gene due to links with basal ganglia calcification^23^ (Figures 2A and 4B, and Table S6). Protein:protein interaction analysis^24^ of the 75 genes whose expression was dependent upon the rs11610045 genotype identified a high-confidence interaction (score ≥0.700) network involving THBS1 and PDGFB (Figure 4C). The genes/proteins within this small network are involved in integrin binding and extracellular matrix organization.^25^^,^^26^

Table S6. Genomics England Parkinson disease gene panel

None of the 75 rs11610045-genotype-dependent genes are proximal to rs11610045. However, possible explanations include that the regulatory interactions occur (1) in *trans* through a microRNA-dependent mechanism; (2) as a downstream effect of rs11610045-variant-dependent changes on expression of a transcription factor (TF); or (3) by feedforward or feedback mechanisms through the affected pathway(s). Analysis using miRDB^27^ highlighted several common miRNA targets across the 75 genes, including hsa-miR-182-5p, hsa-miR-664b-3p, and hsa-miR-9-5p, each of which targets 7 of the 75 genes (Table S7).

Table S7. miRDB target prediction analysis for the 75 DEGs listed in supplementary table 6

### Changes in methylation levels in target genes are linked to changes in their expression

**Figure 5:**
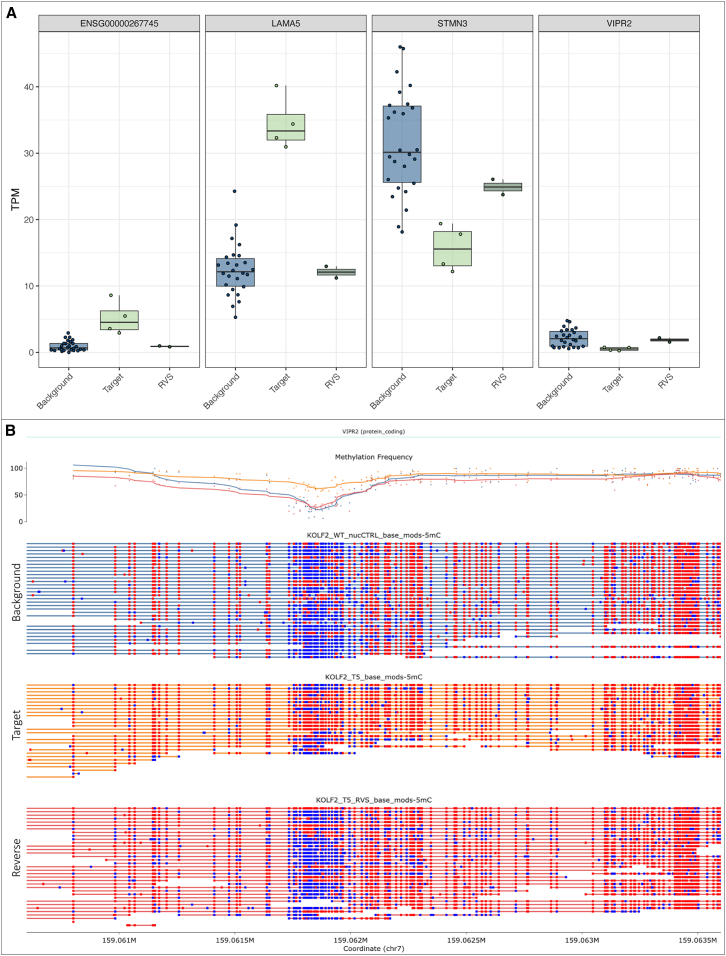
Target genes that reverse expression in an rs11610045-genotype-dependent manner exhibit differential 5 mC methylation profiles (A) Boxplots of TPM (transcripts per million) for a subset of rs11610045-genotype-dependent genes (*ENSG00000267745*, *LAMA5*, *STMN3*, and *VIPR2*). Boxplots show the median (center line), interquartile range (box), and whiskers extending to 1.5× the interquartile range. Individual data points are overlaid. (B) 5 mC differential methylation plots of 3 Kb region located on *VIPR2*, for background, rs11610045, and edit reversal. Methylation profiles for *LAMA5*, *STMN3*, and *ENSG00000267745* are presented in Figures S3A–S3C.

In mammalian genomes, differential methylation of regulatory regions is linked to their activity. In addition to informing on the accumulation of non-specific genetic changes, ONT long-read WGS of the CRISPR-Cas9-edited clones enabled us to explore the 5 mC methylation profiles of the genes whose expression was rs11610045 genotype dependent. Differentially 5 mC methylated regions (*p* < 0.01; Δ change >10%) were identified at the *VIPR2*, *LAMA5*, and *STMN3* genes whose expression was rs11610045 genotype dependent (Table S8)*.* In these three instances, 5 mC methylation across the gene decreased when gene expression increased in response to the rs11610045 genotype (A|A vs. G|G; Figure 5). Notably, for *VIPR2* and *STMN3*, the 5 mC methylation tended toward WT KOLF2.1J levels following reversal of the edit from the G|G to A|A genotype (Figures 5B and S3). For *VIPR2* and *STMN3*, the rs11610045-variant-dependent changes in gene expression that are associated with the differential methylation may result from a change in the expression of a DNA methylase/demethylase. However, the pattern of methylation changes was not ubiquitous across the genes whose expression was rs11610045 variant dependent, consistent with the methylation state having context-dependent functionality.^28^ Context-dependent functionality is further supported by the observation that methylation and expression levels were directly correlated for *ENSG00000267745*, a long non-coding RNA (lncRNA) gene (Figure S3C).

Table S8. Differentially methylated regions in rs11610045 target genes

### Quantitative proteomics identified rs11610045-variant-dependent regulatory proteins

Regulatory regions are read by sequence-specific TFs to control gene activity. The introduction of an SNP into a regulatory region can disrupt or increase the binding of TFs, altering the regulatory potential of the regulatory region. To determine if there are TFs that bind to the regulatory region where the rs11610045 SNP is found and if the introduction of the alternative genotype (G|G) impacts TF binding, we performed affinity purification followed by mass spectrometry. To identify proteins that differentially bind to the rs11610045 A|A or edited G|G genotypes, oligonucleotides (50 bp) centered on either the rs11610045 reference or alternate alleles were incubated with KOLF2.1J iPSC nuclear extract (Figure 2B), and enriched proteins were quantified by mass spectrometry. This analysis identified TCF7L1 as a TF that binds to the rs11610045 A|A genotype more weakly compared to the rs11610045 G|G allele (Table S9). There was also another TF, CEBPZ, that showed the opposite trend. These TFs potentially contribute to the altered gene regulatory activity of rs11610045 and have been associated with neurogenesis and neurological disorders.^29^^,^^30^^,^^31^ In addition, CCDC86, NOL10, YTHDC2, and SRSF7 were also found enriched on the rs11610045 A|A genotype, they are associated with RNA processing and cell division, and their disruption might also contribute to the difference in gene activity.^32^^,^^33^

Table S9. Raw CSV output file for affinity purification followed by mass spectrometry

### Variant-dependent gene regulation differs between iPSC-derived cortical neurons and undifferentiated iPSCs

**Figure 6:**
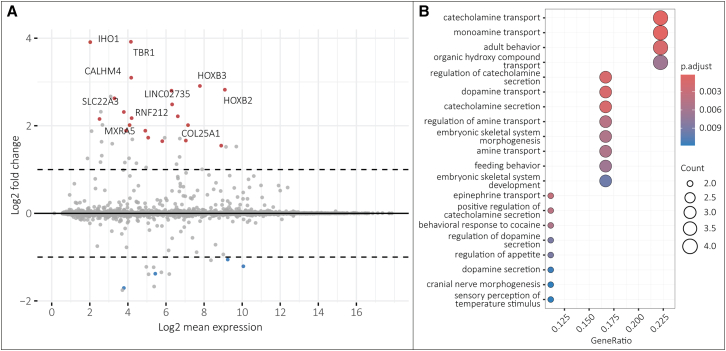
rs11610045 regulates a different set of target genes in iPSC-derived cortical neurons (A) MA plot highlighting the 10 genes whose expression is most significantly associated with rs11610045 genotype in iPSC-derived cortical neurons. Red and blue coloring indicates DEGs with log2FC > 1 and adj. *p* value <0.05. (B) Gene set enrichment analysis for DEGs associated with rs11610045. Benjamini-Hochberg method was used to calculate the adjusted *p* value.

To determine the impact of the introduced PD risk SNP in a more disease-relevant context, we differentiated the WT and edited iPSC lines along a cortical neuronal lineage^34^ (Figure S6). This was not completed for the reverse edit clones. Comparison of the edited iPSC-derived neurons with their respective control lines identified 24 DEGs (log2FC > 1, adj*. p* value <0.05; 20 upregulated and 4 downregulated) associated with the rs11610045 G|G genotype (Figure 6A and Table S10). Only one gene, *PAX5*, overlapped with those differentially expressed in the undifferentiated iPSC lines. Pathway analysis of the 24 genes revealed enrichment for catecholamine, monoamine, and dopamine transport pathways, driven by *CARTPT*, *HTR2A*, *SLC22A3* and *OPRK1* (Figure 6B and Table S11). Given the relatively small number of DEGs, these enrichments should be interpreted with caution, as they are driven by a limited subset of genes. It is perhaps unsurprising that the target gene set for rs11610045 differs depending on cell type and developmental state, given the well-established cell-type specificity of gene regulatory networks. Together, these findings indicate that while the variant exerts regulatory effects in both contexts, the downstream targets and pathways are modulated in a state- and/or cell-type-dependent manner.

Table S10. rs11610045 DEGs compared to all other targets in iPSC-derived cortical neurons (log2FC > 0; *p* > 0.05)

Table S11. Gene set enrichment analysis of the 24 differentially expressed genes associated with rs11610045 in cortical neurons

## Discussion

Here, we utilized an integrative approach to identify the allele-specific impact of the PD-associated rs11610045 on gene regulation. Our findings confirm the hypothesis that this variant is an allele-specific enhancer, capable of impacting gene expression in a context-dependent manner. Although rs11610045 has been associated with PD in genetic studies, the present work investigates its regulatory function rather than directly modeling neurodegeneration. As with all germline variants, its contribution to disease risk is likely shaped by environmental exposures and interactions that occur over a lifetime. As such, it is important to understand the impacts of the disease-associated variant in the undifferentiated developmental state and its potential impacts on future cell states. Our results demonstrated multiple specific impacts for rs11610045 and confirm that although a single base is responsible, the association with PD is complex and likely to be mediated by multiple regulatory targets.

Arguably, the restoration of target gene expression upon reversal of the edited rs11610045 genotype definitively confirms a causal role for the SNP in gene expression, as opposed to an off-target or bystander effect.^35^ It is notable that the target genes of rs11610045 are all located on different chromosomes to the variant itself. There is currently relatively little evidence linking the affected genes to PD pathogenesis. However, this does not diminish the potential impacts. For example, *THBS1*, which shows rs11610045-genotype-specific expression, has been linked to PD in clinical studies exploring gene expression^36^ and has also been shown to interact with *LRRK2* to promote ER stress.^22^ Further, *CEL* has previously been associated with early-onset PD in a Finnish GWA study, and several studies have highlighted a potential role of lipid metabolism in neurodegenerative disorders.^21^^,^^37^ Finally, the vasoactive intestinal peptide receptor 2 (*VIPR2*) has been implicated in PD through both alterations in expression levels in submucosal biopsies^38^ and its neuroprotectant role in dopaminergic neurodegeneration.^39^

Thus, while the evidence that links these genes to PD development is weak, the observation that they are differentially expressed in an rs11610045-allele-specific manner within the undifferentiated KOLF2.1J cell line—which can be differentiated into complex brain organoids^40^^,^^41^—provides an experimental avenue for future investigation.

Consistent with the well-established cell-type and developmental-state specificity of gene regulation, we identified a distinct set of rs11610045 target genes in iPSC-derived cortical neurons compared to the undifferentiated iPSC state. *PAX5*, the only DEG shared between both undifferentiated iPSCs and iPSC-derived cortical neurons, encodes a TF that has previously been implicated in PD-associated gene regulatory networks.^42^ It is plausible that the consistent modulation of *PAX5* across both cellular contexts implicates it as a core regulatory target of rs11610045, potentially acting upstream of some of the other observed transcriptional changes. At the pathway level, the neuronal DEGs showed enrichment for catecholamine, monoamine, and dopamine transport pathways—processes central to neurotransmitter handling and neuronal function. Although the number of DEGs is relatively small, the enrichment of these pathways is notable given their direct relevance to PD pathology. Among these, genes such as *HTR2A*, *SLC22A3*, and *OPRK1* have been previously implicated in synaptic transmission and dopaminergic regulation, suggesting that rs11610045 may influence neuronal physiology through modulation of neurotransmitter release. While further validation in dopamine neuron and glial models is required, these findings point to a potential mechanism whereby rs11610045 contributes to PD risk via cell-type-specific regulation of neurochemical signaling pathways.

### Limitations of the study

A well-documented limitation of CRISPR technology is the introduction of unwanted non-specific edits. In this study, we utilized Cas9 RNP, and not a plasmid-based system, as the RNP is immediately active upon delivery to the cell when the donor template is at maximal levels. Additionally, the Cas9 RNP is degraded within 24 h, limiting the potential for on-going modifications. While this method was successful in achieving low numbers of classically described off-target edits, we did identify accumulated unintended “genetic variation” (SNVs and indels) that likely occurred during clonal selection and cell growth (mutational load). Of note, ONT long-read WGS enabled us to computationally remove any DEGs that were likely targets of this accumulated variation. Our reliance on *in silico* subtraction of DEGs due to the presence of accumulated mutations might mean we have missed subtle off-target effects. As such, we cannot say with complete certainty that the identified DEGs result from the desired edit as opposed to other introduced variation.

Exploring allele-specific regulatory effects within undifferentiated iPSCs is problematic due to cellular heterogeneity. To minimize this, all cells were constantly maintained and passaged only during the log phase. Nguyen et al. previously completed scRNA-seq of human iPSCs and determined four sub-populations within their iPSC population that represented different states of pluripotency: (1) core; (2) proliferative; (3) early primed; and (4) late primed.^43^ Based on our own bulk RNA-seq data, we observed that while our clones are still considered pluripotent (as indicated by consistent expression of *NANOG*, *SOX2*, and *OCT4*), some are within different sub-populations and are at “later,” more primed stages. It is intriguing to speculate on whether the introduced variants are driving the cells toward a primed state or whether the different states are due to technical variability. However, it seems the first explanation may be more plausible given that all replicates of a particular target cluster together, rather than seeing sub-population variation within a single target. Thus, the use of bulk RNA-seq, which averages measurements, can be questioned, as it fails to provide insight into the heterogeneity of cell states that represent pluripotency. Future studies that incorporate scRNA-seq or other new technologies (e.g., Evercode combinatorial barcoding [Parse Biosciences]) would refine our observations by addressing these limitations.

An additional limitation is that RNA-seq of iPSC-derived cortical neurons was performed for the WT and forward-edited lines only. Although inclusion of reverted clones at the neuronal stage would further strengthen causal inference, these experiments were beyond the scope of the present study. Importantly, neuronal differentiation and marker expression were comparable across genotypes, and transcriptomic changes observed support a variant-dependent regulatory effect.

While some eQTL interactions are seen consistently across different cell states and types, many *cis-* and *trans-*interactions are cell-type- and developmental-state-specific.^44^^,^^45^ It is, therefore, intriguing to dissect the role of the identified interactions in the iPSCs and understand how (dys-)regulation of these genes may impact later states. Given that many *cis*- and *trans*-regulatory interactions are highly cell-type- and developmental-state-specific,^44^^,^^45^ future studies should incorporate high-resolution chromatin conformation approaches in both edited and reverted lines to further delineate long-range regulatory interactions. Similarly, experiments that perturb the methylation machinery, and incorporate more detailed temporal analyses, are required to assert causal relationships between rs11610045, methylation changes, and alterations to gene expression.

As GWA studies grow in number and scale, the number of risk variants linked to any trait or disease continues to increase. As such, there is a growing need to up-scale functional studies that enable the translation of risk into mechanistic understandings. Our approach shows the many steps needed to identify the functional targets of non-coding variants and reveals some of their complexities. Moving forward, base editing within iPSC-derived cell lines will enable a deeper functional understanding of the impact of GWAS variants in disease-relevant contexts.

## Resource availability

### Lead contact

Requests for further information and resources should be directed to and will be fulfilled by the lead contact, Sophie L. Farrow (sophie.farrow@dpag.ox.ac.uk).

### Materials availability

Isogenic iPSC lines generated in this study are available from the lead contact upon reasonable request and completion of a materials transfer agreement.

### Data and code availability

• RNA-seq and long-read WGS data have been deposited in the NCBI Sequence Read Archive (SRA) and are publicly available as of the date of publication. AP-MS data are available via the PRIDE repository and are publicly available as of the date of publication. Accession numbers are listed in the key resources table.
• All original code has been deposited in appropriate repositories and is available at https://github.com/sfar956/PD_iPSC_Tx.git. R Studio v.4.2.1 was used for data analyses and visualization.
• Summary data are provided in the supplementary tables.
• Any additional information required to reanalyze the data reported in this paper is available from the lead contact upon request.

## Acknowledgments

J.M.O’S., A.C., R.G., and S.L.F. were funded by the 10.13039/100000864Michael J. Fox Foundation – grant ID 021131 to J.M.O’S. A.A.C. received grant funding from the 10.13039/100015539Australian Government. S.L.F. received funding from 10.13039/501100000304Parkinson’s UK – grant ID AVR03420. S.G. and D.N. were funded by the Dines Family Charitable Trust. We would like to acknowledge Mark Cookson and colleagues for providing methodological guidance and advice. We would also like to thank the following: New Zealand e-Science Infrastructure (NeSI) for providing high-performance computing; Peter Tsai for providing valuable input for the analysis of ONT long-read WGS data; William Skarnes and the Jackson Laboratory for provision of the KOLF2.1J iPSC line; and the Alzheimer disease data initiative for provision of the associated KOLF2.1J iPSC line sequencing data.

## Author contributions

J.M.O’S., A.A.C., and S.L.F. conceived and led the study. S.L.F., J.M.O’S., R.S.G., and A.A.C. designed the experimental procedures, and S.L.F. conducted all experimental procedures with the exception of the AP-MS, which was conducted by R.S.G. and I.S.A. S.L.F. completed all data visualizations. S.G. developed the RNA-seq analysis script and performed data analyses. D.N. completed off-target and methylation analyses of the ONT long-read WGS data. R.S.G. and A.A.C. advised on the study. S.L.F. and J.M.O’S. wrote the manuscript. All authors commented on the manuscript.

## Declaration of interests

The authors have no conflicts of interest to disclose.

## STAR★Methods

### Key resources table

**Table:** 

REAGENT or RESOURCE	SOURCE	IDENTIFIER
**Antibodies**		

B-III tubulin; chicken	Novus	NB100-1612; RRID:AB_10000548
MAP2; mouse	Novus	NBP2-25156AF350; RRID:AB_3273979
SNAP25; rabbit	Novus	NBP1-33714; RRID:AB_2192187

**Biological samples**		

KOLF2.1J iPSC line	The Jackson Laboratories	JIPSC001000 | https://www.cell.com/cell-stem-cell/fulltext/S1934-5909(22)00451-9
Isogenic edited KOLF2.1J iPSC lines (rs11610045 A|A and G|G)	This study	Available upon request

**Chemicals, peptides, and recombinant proteins**		

StemFlex Medium	Thermo Fisher Scientific	A3349401
Synthemax II-SC substrate	Corning	3535
ReLeSR	STEMCELL Technologies	5872
Accutase	Thermo Fisher Scientific	A1110501
RevitaCell Supplement	Thermo Fisher Scientific	A2644501
Alt-R HDR Enhancer V2	IDT	10007921
Alt-R S.p. HiFi Cas9 Nuclease V3	IDT	1081061
Platinum II HS Master Mix	Thermo Fisher Scientific	14000013
Monarch PCR & DNA Cleanup Kit	NEB	T1030L
RNeasy Plus Mini Kit	Qiagen	74104
KnockOut DMEM/F12	Thermo Fisher Scientific	12660012
N2 Supplement	Thermo Fisher Scientific	17502048
Non-essential amino acids	Thermo Fisher Scientific	11140050
GlutaMAX	Thermo Fisher Scientific	35050–061
BrainPhys Medium	STEMCELL Technologies	5790
N21-MAX	R&D Systems	AR008
GDNF	PeproTech	450–10
BDNF	PeproTech	450–02
NT-3	PeproTech	450–03
Chroman I	MedChemExpress	HY-15392

**Critical commercial assays**		

Alt-R HDR donor oligos	IDT	Custom
Protein BR Assay Kit	Thermo Fisher Scientific	A50668
Ligation Sequencing Kit (ONT)	Oxford Nanopore Technologies	SQK-LSK114
NEBNext Companion Module	NEB	E7180L

**Deposited data**		

RNA-seq data	NCBI SRA	PRJNA1251319
ONT long-read WGS data	NCBI SRA	PRJNA1251319
AP–MS data	PRIDE	PXD065108

**Experimental models: Cell lines**		

KOLF2.1J iPSC line	The Jackson Laboratories	JIPSC001000
Isogenic edited KOLF2.1J iPSC lines (rs11610045 A|A and G|G)	This study	Available upon request

**Oligonucleotides**		

sgRNAs targeting rs11610045	This study	See Table S1
ssODN repair templates	IDT	See Table S1
PCR primers	This study	See Table S1
AP–MS oligonucleotides	Microsynth	See Table S1

**Recombinant DNA**		

PB-TO-hNGN2	Addgene	172115
K13-EF1a-transposase plasmid	iNDI	Collaborator supplied

**Software and algorithms**		

STAR (v2.7.9a)	Dobin et al.	https://github.com/alexdobin/STAR
Subread/featureCounts (v2.0.3)	Liao et al.	http://subread.sourceforge.net
DESeq2 (v1.42)	Love et al.	https://bioconductor.org/packages/DESeq2
MultiQC (v1.13)	Ewels et al.	https://multiqc.info
Minimap2 (v2.26)	Heng Li	https://github.com/lh3/minimap2
EPI2ME wf-alignment	ONT	https://github.com/epi2me-labs/wf-alignment
EPI2ME wf-somatic-variation	ONT	https://github.com/epi2me-labs/wf-somatic-variation
modkit (v0.2.3)	ONT	https://github.com/nanoporetech/modkit
CRISPR_Tx github repository	Farrow et al.	https://github.com/sfar956/PD_iPSC_Tx
MaxQuant	Cox & Mann	https://www.maxquant.org
Cas-OFFinder	Bae et al.	http://www.rgenome.net/cas-offinder/
CRISPOR	Concordet & Haeussler	http://crispor.tefor.net
Wellcome Sanger Institute Genome Editing tool	Wellcome Sanger Institute	https://wge.stemcell.sanger.ac.uk

**Other**		

Amaxa 4D Nucleofector	Lonza	N/A
Nucleocuvette strips (100 μL)	Lonza	V4XP-3024
PromethION Flow Cells (R10.4.1)	ONT	FLO-PRO114M
PromethION 2 Solo	ONT	N/A

### Experimental model and study participant details

The KOLF2.1J iPSC line^46^^,^^47^ was obtained from Jackson laboratory (https://www.jax.org/jax-mice-and-services/ipsc). As previously described,^47^ the KOLF2.1J iPSC line is well-characterised and has been identified as a robust, reference iPSC-line. The KOLF2.1J line is derived from a donor of European ancestry and is genetically male. In-house long-read whole genome sequencing was performed on both control and edited cell-lines to confirm maintenance of normal karyotypes (see methods). All cell lines were routinely tested and confirmed negative for mycoplasma contamination.

### Method details

#### KOLF2.1J iPS cell culture and single base editing

##### KOLF2.1J cell culture

Cell culture and gene editing methods used were based upon previously published methods developed by William Skarnes and colleagues specifically for the KOLF2.1J line.^9^^,^^48^ Briefly, KOLF2.1J cells were maintained (37°C; 5% CO_2_) in Stemflex media (Thermo Fisher #A3349401) on Synthemax II-SC substrate (Corning #3535; Figure S4). Cells were colony-passaged every 4–5 days at ∼70–80% confluency using ReLeSR. Cell viability was measured using the Countess II FL Automated Cell Counter and maintained at >90% live cells.

##### sgRNA and ssODN design

Single guide RNAs (sgRNAs) were designed for the KOLF2.1J rs11610045 A|A genotype (WT) using the Wellcome Sanger Institute Genome Editing tool (WGE; https://wge.stemcell.sanger.ac.uk/), and sequences were confirmed against the KOLF2.1J genome (Figure S1). Additional sgRNAs were designed for 6 other targets (Table S1).

CRISPOR^18^ and IDT design tools were used for *in silico* predictions of efficiency and off-target effects. In general, sgRNAs were selected based on low predicted off-target effects and with the target cut-site near to the PAM site. A GC% of between 40 and 70% was also desirable. Synthetic sgRNAs (synthesised with 2′ O-Methyl and 3′ phosphorothioate end modifications) were purchased from Synthego and resuspended in 1× TE buffer to a concentration of 100 μM. Single-strand DNA oligonucleotides (ssODNs) were designed based on previous analyses^48^ to achieve maximum efficiency (100 nt length; complementary to target strand; asymmetric arms [35/65 nt around target]). ssODNs were purchased from IDT (Alt-R modified HDR donor oligos) and resuspended in D-PBS to a concentration of 200 pmol/μL sgRNA and ssODN sequences are detailed in Table S1.

##### *In vitro* cas9 sgRNA assay

Efficiency of sgRNAs was tested using an *in vitro* assay prior to nucleofection in the KOLF2.1J cells. For rs11610045, primer pairs were designed to amplify an ∼1000bp genomic region, centered on the target site (Table S1). The same was undertaken for the additional targets (Table S1). 20 μL PCR reactions were prepared with the following reagents: 10uL Platinum II HS master mix (2×), 0.4uL 10 μM forward and reverse primers, 0.5uL KOLF2.1J purified genomic DNA (200 ng/μL), 8.7 μL dH_2_O. 35 PCR cycles were completed (94°C 15s; 60°C 15s; 68°C 15s). For the *in vitro* assay, the following reagents were prepared and incubated at 25°C for 10 min: 1× NEB r3.1 restriction enzyme buffer; 500 ng cas9 nuclease (IDT Alt-R S.p. HiFi Cas9 Nuclease V3 #1081061); 400 ng sgRNA; dH_2_O to 20 μL. Following incubation, ∼200 ng of the respective purified PCR product was added to each reaction for a further 30 min incubation at 37°C. 1 μL proteinase K was then added to each reaction and incubated at room temperature for 10 min. The digested products were then run on a 1.5% agarose gel for 2 h, and cutting efficiency of the sgRNAs assessed (Figure S12).

##### Nucleofection

Nucleofection was carried out using 100 μL nucleocuvettes (Lonza #V4XP-3024) and an Amaxa 4D nucleofector (Primary cell P3 program; pulse code CA-137). For each target, 10 μg Cas9 RNP and 8 μg sgRNA (1:4 cas9:sgRNA molar ratio) were mixed and incubated at room temperature for 30-45 min. During incubation, a single cell suspension was prepared by treating one well (of a six-well plate; number of wells dependent on number of nucleofections being carried out) with Accutase and incubating at 37°C for 7 min. Cells were then pelleted and resuspended in Stemflex for counting. 8×10^5^ cells were pelleted per reaction. While pelleting the cells, 200 pmol ssODN was added to the cas9:sgRNA mix. The cell pellet was then resuspended in 100 μL complete P3 solution (Lonza) and then mixed with the cas9:sgRNA:ssODN mix for nucleofection. Immediately following nucleofection, cells were transferred to one well of a six well plate containing the following reagents: Stemflex media; 1× RevitaCell (Thermo Fisher #A2644501); 1 μM Alt-R HDR enhancer V2 (IDT #10007921). The cells were then incubated at 32°C; 5% CO_2_^9^ for 48 h before returning to 37°C. After 24hrs, the media was replaced with Stemflex only, and then every other day until reaching 80% confluency.

##### Single cell selection

Upon reaching 80% confluency, cells were dissociated to single cells with Accutase (37°C for 7 min) and were split into three sets: (1) cells were frozen (Knockout Serum Replacement +10% DMSO); (2) DNA extracted from ∼50,000 cells for Sanger sequencing (screening the cell pool was used as a rough guide to determine success of the editing before continuing with single cell selection); (3) 1,500 cells plated on a vitronectin-coated 10 cm plate containing 10 mL Stemflex with 1× RevitaCell. Following confirmation of a successful edit (Sanger seq), cells were maintained in the 10 cm plate for ∼10 days until iPSC colonies were visible. Single colonies (usually ∼24) were then manually picked and grown in duplicate in a 96-well plate. DNA was prepared from the cells grown for sequencing using the Platinum Directed PCR Universal Master Mix (Thermo Fisher #A44647100) and ∼1000bp fragments were amplified as described earlier. PCR amplified products were purified using the Monarch PCR & DNA Cleanup kit (NEB #T1030L) and Sanger sequencing completed (Figure S3). Clones containing the desired edit were then expanded and stocks frozen.

##### CRISPR reversal

Following successful introduction of the desired edit, we used the same cas9 protocol to edit rs11610045 clones with the G|G genotype (clones A4 and A5) back to wild-type A|A genotype to enable us to determine if gene expression levels reverted. The same protocol was used as described above, with updated sgRNAs and ssODNs (Table S1, column S).

#### Cortical neuron differentiation

##### Piggybac-TO-hNGN2 transfection

Protocols developed by Mark Cookson and colleagues were used for the differentiation of the KOLF2.1J cell line (WT and edited) to cortical neurons.^34^^,^^49^ Briefly, the cells were first maintained in E8 medium (Thermo Fisher #A1517001) supplemented with CEPT^50^ on Matrigel coated 6-well plates. Cells were then prepared as previously described and transfected with EF1a-transposase and PB-TO-hNGN2 (Addgene #172115) at a ratio of 1:3 using lipofectamine stem (Thermo Fisher #STEM00003). Positive transfection was confirmed through observation of nuclear BFP signal. Cells were then replated and puromycin selection was started 24-48h later and continued for ∼10 days. Media was changed every other day, and cells were expanded upon reaching ∼70–80% confluency.

##### Cortical neuron induction and maturation

Following stable integration of human NGN2, cells were exposed to doxycycline (2 μg/mL) and maintained in Knockout DMEM/F12 media (Thermo Fisher #12660012) supplemented with N2 (Thermo Fisher #17502048), non-essential amino acids (Thermo Fisher #11140050), glutamax (Thermo Fisher #35050-061) and chroman I (MCE #HY-15392) on Matrigel (Corning #354277) for days 0–3. On day 3 media change, Uridine and Fluorodeoxyuridine was also added to suppress mitotic cells. On day 4, the pre-differentiated neurons were replated for final neuronal maturation on poly-L-ornithine. From days 4–9, the cells were maintained in Knockout DMEM/F12 and Brainphys medium (50:50 [Stem Cell Technologies #05790]) supplemented with N21MAX (R&D systems #AR008), GDNF (Peprotech #450-10), BDNF (Peprotech #450-02), NT-3 (Peprotech #450-03) and doxycycline, with media changes every 3 days. From day 10 onwards, the Knockout DMEM/F12 component was removed from the medium, and media changed every 3–4 days until full maturation at 28 days.

##### Immunocytochemistry cell staining

Cultures grown in 48-well plates were washed with PBS, fixed with 4% paraformaldehyde in PBS for 15 min, and washed three times with PBS-T (PBS with 0.1% Triton X-100). Primary antibodies were prepared in immunobuffer with goat serum. Fixed samples were incubated with the prepared primary antibodies at 4°C overnight. Samples were then incubated with AlexaFluor-labelled secondary antibodies for 3 h at room temperature. Cells were imaged using an EVOS-FL auto imaging system (Life Technologies). Antibodies used in this study: B-III tubulin (1:1000, Novus, #NB100-1612, chicken); MAP2 (1:250, Novus, pre-conjugated #NBP2-25156AF350, mouse); SNAP25 (1:500, Novus, #NBP1-33714, rabbit).

#### RNA extraction, sequencing and data analysis

##### RNA extraction

Following successful introduction of the desired edit and expansion of multiple clones, cells (70–80% confluence) were lysed directly in 24-well plates and processed using the RNeasy+ mini kit (Qiagen #74104), according to the manufacturer’s instructions. RNA integrity (i.e., RIN) was checked using the bioanalyzer, and all samples passed quality control.

##### RNA sequencing

Stranded mRNA library preparation was performed with a minimum of 2000 ng of total RNA. RNA was sequenced using the following parameters: stranded, poly-A enriched mRNA library; 150 paired-end sequencing (Novaseq, 30M reads per sample; Custom Science).

##### RNA sequencing data analysis

RNA-seq reads were quality checked using multiQC (v1.13)^51^ and mapped against the human reference genome GRCh38 using STAR (v 2.7.9a)^52^ aligner software. Following read alignment, we used featurecounts^53^ module from the Subread (v2.0.3) suite to count the number of reads mapping over individual genes. A matrix of gene counts per annotated gene was generated using Gencode project genes (v43) (https://www.gencodegenes.org/). The gene count matrix generated by the featurecounts program was then used as an input for an R package DESeQ2 (v1.42)^54^ for differential gene expression analysis using default settings. We defined differentially expressed genes as those with absolute log2 fold change (log2FC) difference >1 and Bonferroni adjusted *p*-value <0.05.

In our RNA-seq analysis of the undifferentiated iPSC lines we observed significant PC1 variance (77%), which was not explained by batch effect (Figure S5A). Therefore, we characterised the pluripotency states of the iPSC lines based on Nguyen et al. in 2018.^43^ There was relatively little variation in expression levels of key markers of pluripotency (*i.e*., *OCT4* and *SOX2*) across all lines (Figure S5B). However, analysis of the expression levels of key marker genes for specific pluripotency stages (i.e., *IDO1, NANOG, UTF1* and *ZIC1*) identified two sub-groups of clones within the RNA-seq data. Those clones with low expression of *ZIC1, UTF1* and *NANOG* (and higher expression of *IDO1*) are deemed to be in the core pluripotent state (gray color), while those with high expression of *ZIC1, UTF1* and *NANOG* are in the proliferative or primed state of pluripotency (Figures S5C and S5D). Fundamentally, all the cell lines were analyzed in the pluripotent state, but minor nuances show that there is heterogeneity even within a seemingly homogeneous population. This heterogeneity is accounted for in our analysis by treating all clones across pluripotency states as “background” when assigning differentially expressed genes for rs11610045 genotype clones.

#### Affinity purification mass spectrometry

Oligonucleotides for affinity purification (Table S1, column Y) were ordered from Microsynth with a biotin moiety added to the forward primer, suspended (100 μM), and then annealed in a PCR thermocycler ([95°C, 5 min] × 1; [95°C, −0.1°C, 3 s] × 800; 4°C, hold) to give a final concentration of 25 μM of annealed oligo. Oligos were then diluted in DNA binding buffer (DBB: 1 M NaCl, 10 mM Tris (pH 8.0), 1 mM EDTA, 0.05% NP-40) to a 2 μM concentration in a 200-μL volume per replicate. Streptavidine-Sepharose bead slurry (20 μL per replicate, Cytiva, 17511301) was washed once with PBS +0.1% NP-40 (1 mL), the beads collected by centrifugation (2000g, 2 min, 4°C), and the supernatant removed. Then, the beads were washed once with DBB, suspended in the same volume DBB, and added to the diluted oligos. Oligos were bound to streptavidin beads for 30 min at 4°C on a rotating wheel. Samples were then washed once with DBB (1 mL) and twice with protein binding buffer (PBB: 150 mM NaCl, 50 mM Tris, pH 8, 0.25% NP-40, 1 mM Tris(2-carboxyethyl) phosphine (TCEP) and complete protease inhibitor (Roche)).

KOLF2.1J cells (100 × 10^6^) were harvested using ReLeSR and nuclei were extracted by suspending in 5 mL of Buffer A (10 mM HEPES (pH 7.9), 10 mM KCl, 10 mM EDTA, 0.5% NP-40, 1 mM DTT and complete protease inhibitor (Roche)) and incubating on ice for 10 min with vortexing at maximum speed every 2 min for 10s. Nuclei were collected by centrifugation (800*g*, 10 min, 4°C), and the supernatant was carefully removed using a vacuum. Nuclei were disrupted in 750 μL Buffer B (20 mM HEPES (pH 7.9), 400 mM NaCl, 1 mM EDTA, 1% glycerol, 1 mM DTT, complete protease inhibitor (Roche)) by pipetting up and down with a 1-mL pipette until it was possible to pipette them up and down with a 200-μL tip, this was done on ice. Disrupted nuclei were placed into a vortex block in a cold room, vortexed at maximum speed for 10 s, and then incubated at mid-speed (around 5) for 2 h at 4°C, with vortexing at maximum speed for 10 s every half an hour. Samples were centrifuged (800*g*, 10 min, 4°C), and the supernatant was collected. The protein quantity was measured by Qbit using Protein BR Assay Kit (Invitrogen, A50668). For each affinity purification,^55^ KOLF2.1J nuclear extract (150 μg) was diluted in a 500 μL final volume in PBB and added to the oligo-bound streptavidin beads. Samples were incubated for 2 h at 4°C with slow rotation. Samples were washed four times with washing buffer (150 mM NaCl, 100 mM ammonium bicarbonate) and twice with PBS. Beads were suspended in elution buffer (50 μL, 2 M Urea, 100 mM Tris (pH 8.4), 10 mM DTT) and incubated for 20 min at room temperature with agitation (1250 rpm), then frozen at −20°C ready for mass spectrometry analysis.

##### Affinity-purification mass spectrometry analysis

Following the affinity purification, IAA was added (50 mM) and incubated in the dark at room temperature with agitation (10 min, 1250 rpm). Proteins were digested with Trypsin (150 ng, Thermo Fisher) and LysC (100 ng, Wako Chemicals) at RT with agitation (2 h, 1250 rpm). Beads were collected by centrifugation (2000g, 2 min), and the supernatant was transferred to a LoBind tube. Beads were suspended in elution buffer (50 μL), incubated at room temperature with agitation (5 min, 1250 rpm), collected (2000g, 2 min), and the supernatant added to the LoBind tube. Trypsin (100 ng) was added and cleaved overnight with intermittent agitation (37°C, 1250 rpm for 5 min per hour). The generated peptides were acidified with 1 μL of 20% TFA and light and medium dimethyl labeling was done to allow to combine the wild-type allele with the alternative allele for simultaneous measurement. The samples were analyzed by capillary liquid chromatography-tandem mass spectrometry connected to a Orbitrap Fusion (Thermo Fisher Scientific).

Protein identification and relative quantification of the proteins was done with MaxQuant. Andromeda search engine was used to search the spectras against the Homo Sapiens UniProt database as reference. Common contaminants and highly abundant background proteins were removed. Protein groups were filtered to keep only ones that were identified with at least two peptides, as well as the ones with one or more unique peptides. Protein intensities were log_2_-transformed, and variability across replicates was calculated using the coefficient of variation (CV). Proteins with a CV above 10% were excluded to ensure reproducibility. Subsequently, log2-transformed intensities of light-labelled (A|A) samples were compared to the medium-labelled (G|G) samples. To identify significantly enriched proteins, a two-sample paired *t* test was performed, and a minimum log-fold change of 1/-1 was required. Processed mass spec data is in Table S9.

#### ONT long-read DNA sequencing

##### High molecular weight (HMW) DNA extraction, library preparation and sequencing

HMW DNA was extracted from KOLF2.1J cell lines using the Monarch HMW DNA extraction kit (NEB #T3050), according to the manufacturer’s instructions. ∼2-3×10^6^ cells were used as starting input. Following elution of HMW ^12^DNA, sequencing libraries were prepared using the ligation sequencing kit (ONT #SQK-LSK114), and NEBnext Companion Module (#E7180L), following the manufacturer’s instructions. Samples were loaded on PromethION flow cells (R10.4.1) and sequenced with the PromethION 2 (P2) solo devices (Oxford Nanopore Technologies [ONT]) using Kit 14 chemistry (with the high accuracy model [dna_r10.4.1_e8.2_400bps_hac@v4.2.0]) and MinKNOW v23.07.8 (Oxford Nanopore Technologies [ONT]) to a depth of ≥30×.

##### Sequence data analysis, off-target characterisation, and DNA methylation analysis

The resulting FASTQ files, with a Phred quality score (Q score) > 9, were aligned to the GRCh38 reference genome using minimap2 (v2.26) (citation) as implemented in EPI2ME Labs’ wf-alignment pipeline (https://github.com/epi2me-labs/wf-alignment; v0.5.2). We employed the EPI2ME Labs’ wf-somatic-variation pipeline (https://github.com/epi2me-labs/wf-somatic-variation; v1.1.0) to identify CRISPR/Cas9-mediated off-targets, together with other genetic variations (including single nucleotide polymorphisms [SNPs], small insertions and deletions [indels], and structural variations [SVs]) arising *de novo*, from paired KOLF2.1J wildtype/edited cell line BAM files for a single sample (*i.e*., the KOLF2.1J line treated as the normal and the edited lines treated as tumors; see https://github.com/epi2me-labs/wf-somatic-variation; v1.1.0). We used custom scripts to process, filter and visualise the identified variants.

For methylation analysis, raw ONT signal data stored in POD5 files (https://github.com/nanoporetech/pod5-file-format) was base called (Dorado v0.5.0) using the high accuracy DNA base modification model (dna_r10.4.1_e8.2_400bps_hac@v4.2.0_5mCG_5hmCG@v2) to identify modified bases, specifically 5-methylcytosine (5 mC). Subsequently, the modified BAM files were aligned to the GRCh38 reference genome using minimap2 (v2.26) (citation) as implemented in EPI2ME Labs’ wf-alignment pipeline (https://github.com/epi2me-labs/wf-alignment; v0.5.2). To generate comprehensive genome-wide quantification of modified and unmodified bases, we employed modkit version 0.2.3 (https://github.com/nanoporetech/modkit) and results exported to bedMethyl format files. Finally, differentially methylated regions (DMRs) between samples were identified and annotated using the ont-methylDMR-kit pipeline as detailed in https://github.com/NyagaM/ont-methylDMR-kit.

### Quantification and statistical analysis

All statistical analyses were performed in R (version 4.2.1). Statistical tests were selected as appropriate for each dataset and are specified in the corresponding Methods sections and figure legends. Multiple testing correction was performed where applicable, and adjusted *p* values <0.05 were considered statistically significant. For differential analyses, significance thresholds were set at adjusted *p* < 0.05 and |log2FC| > 1, unless otherwise stated.
