# Supplementary material for: Dissecting genotype-specific effects of disease-associated genetic variants
Source: iScience. 2026 Jun 1;29(6):116143. doi: 10.1016/j.isci.2026.116143 (PMC13253082; doi:10.1016/j.isci.2026.116143)

iScience, Volume *29*

Supplemental information

Dissecting genotype-specific effects

of disease-associated genetic variants

Sophie L. Farrow, Sreemol Gokuladhas, Izlem Su Akan, Denis Nyaga, Antony A. Cooper, Ralph Stefan Grand, and Justin M. O'Sullivan

Supplementary Figure 1: The genotype of rs11610045 was successfully edited from A|A (WT) to G|G (edited). *a) In vitro Cas9 sgRNA efficiency test; b) Sanger sequencing results showing successful introduction of G|G genotype; c) Sanger sequencing results for the 6 PD-SNP clones also generated in this study, and used as background control clones.*

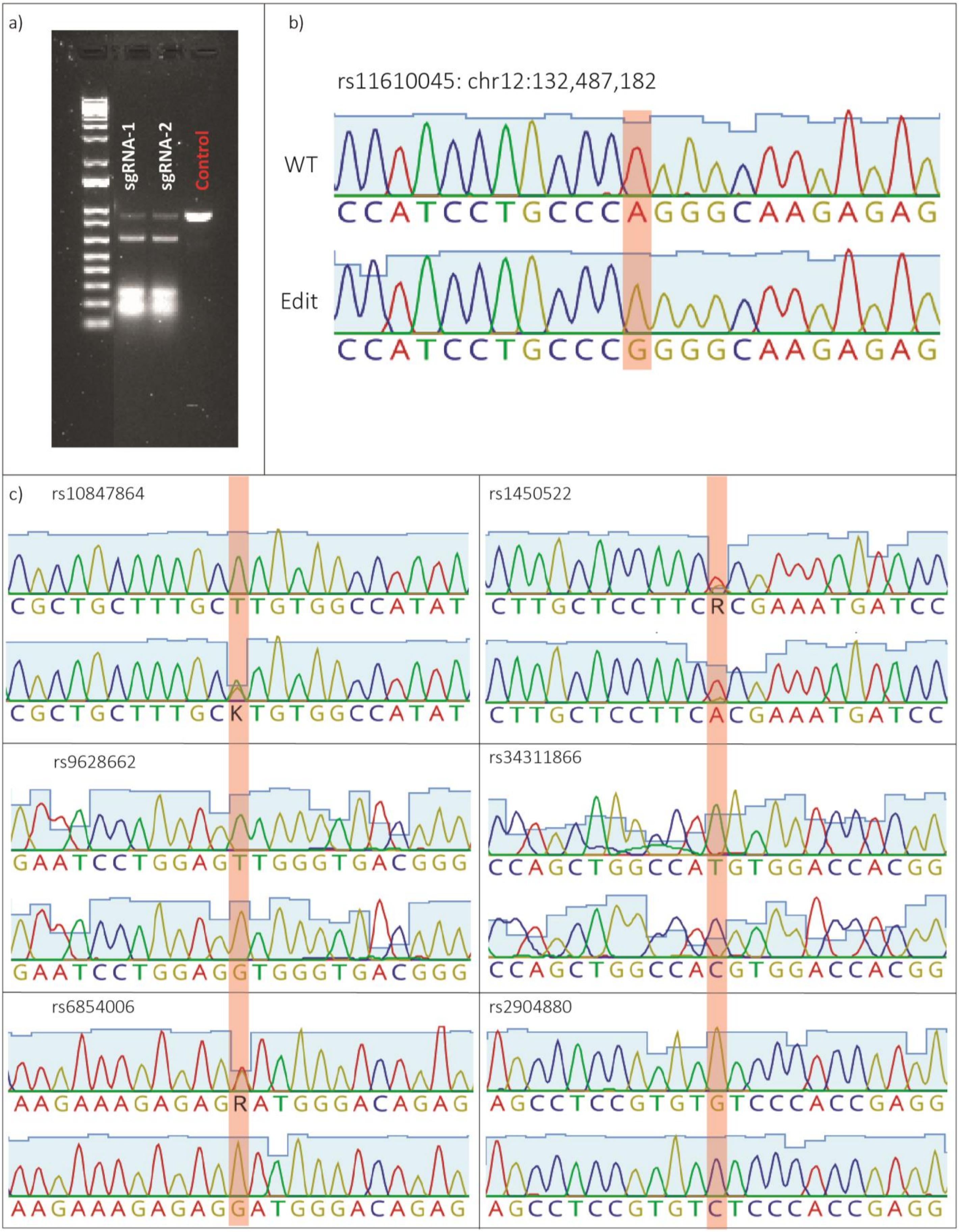

Supplementary Figure 2: Off-target/genetic-variation accumulated and may explain observed expression changes in some genes. a) ADARB2; b) XRCC5; c) NPEPP5

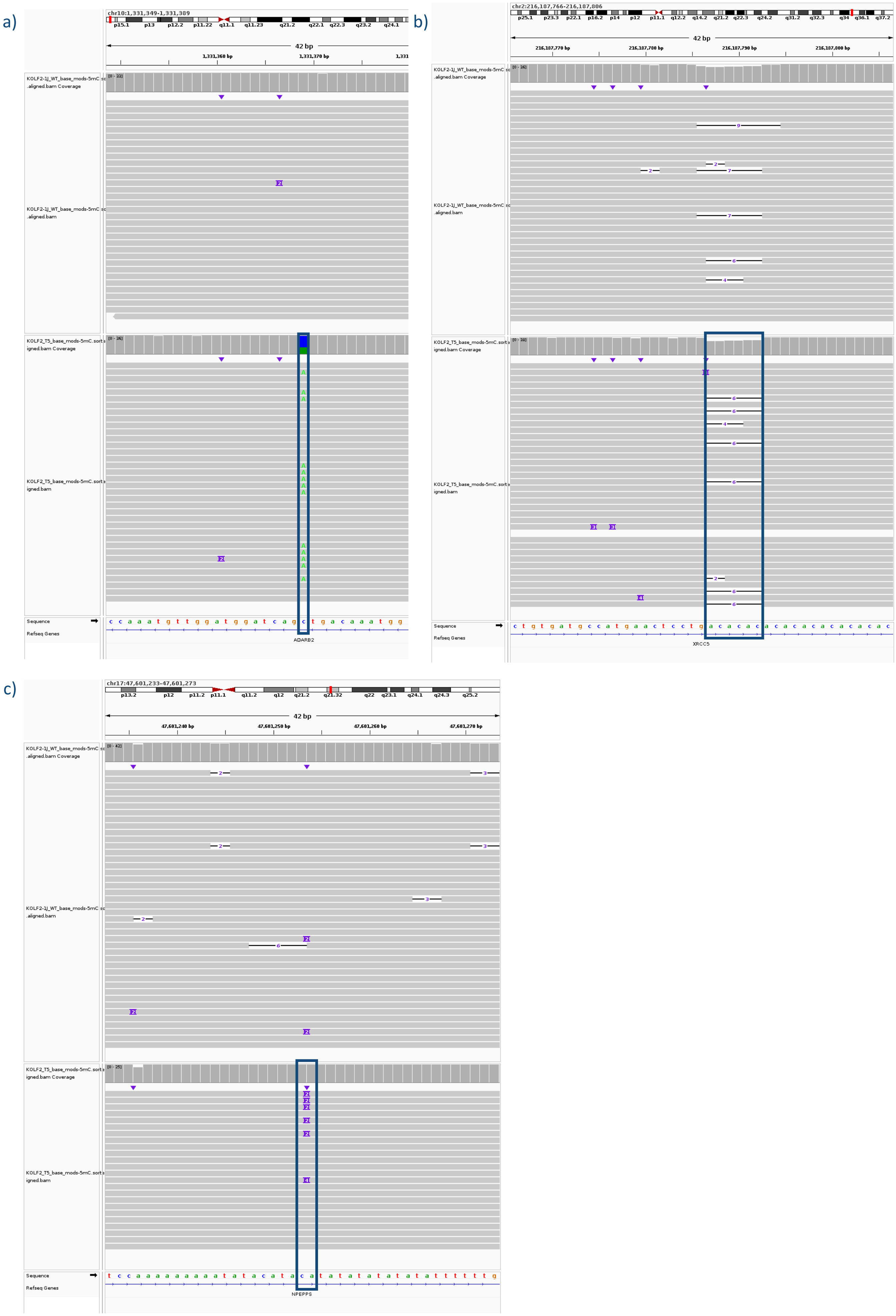

Supplementary Figure 3: a) STMN3; b) LAMA5; and c) ENSG00000267745 expression and 5mC methylation profiles were associated with rs11610045 genotype .

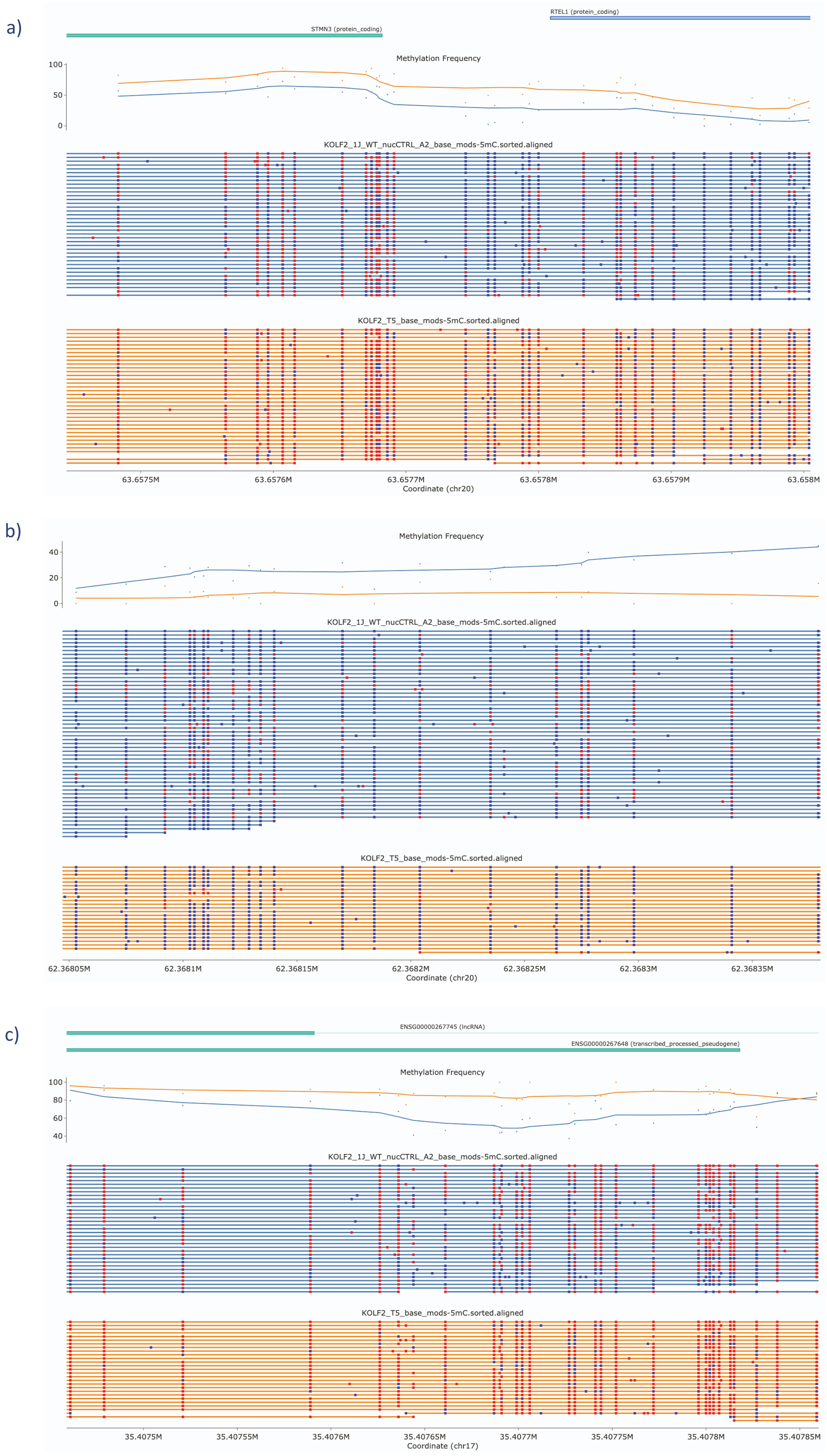

Supplementary Figure 4: Brightfield images of the KOLF2.1J WT iPSC line

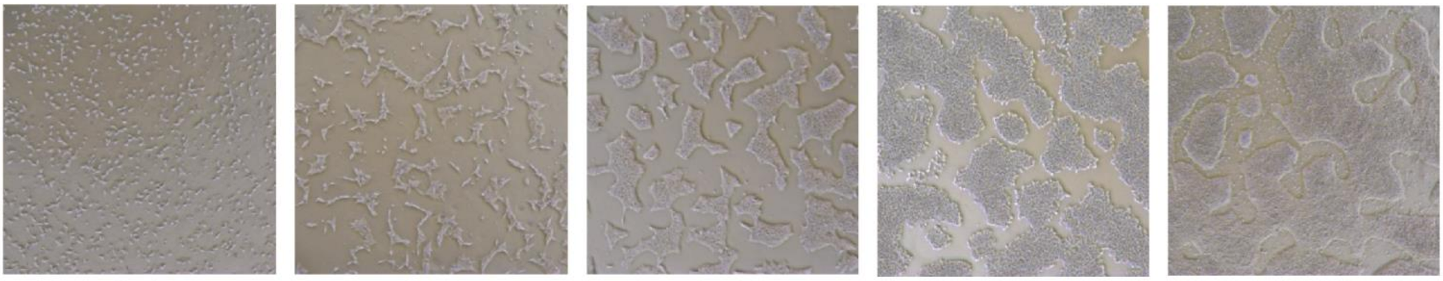

Day 0 | Day 1 | Day 2 | Day 3 | Day 4

Supplementary Figure 5: Variance markers markers of pluripotency indicated variation in the pluripotency state of the sequenced clones. a) PCA plot of background of rs11610045 edited (light green), reversed clones (dark green), and background (navy blue); b) Gene expression levels (TPM) of pluripotency marker genes (Target – rs11610045 edited clones; RVS = edit-reversal clones); c & d) Gene expression levels (TPM) of genes associated with proliferative and primed states of pluripotency.

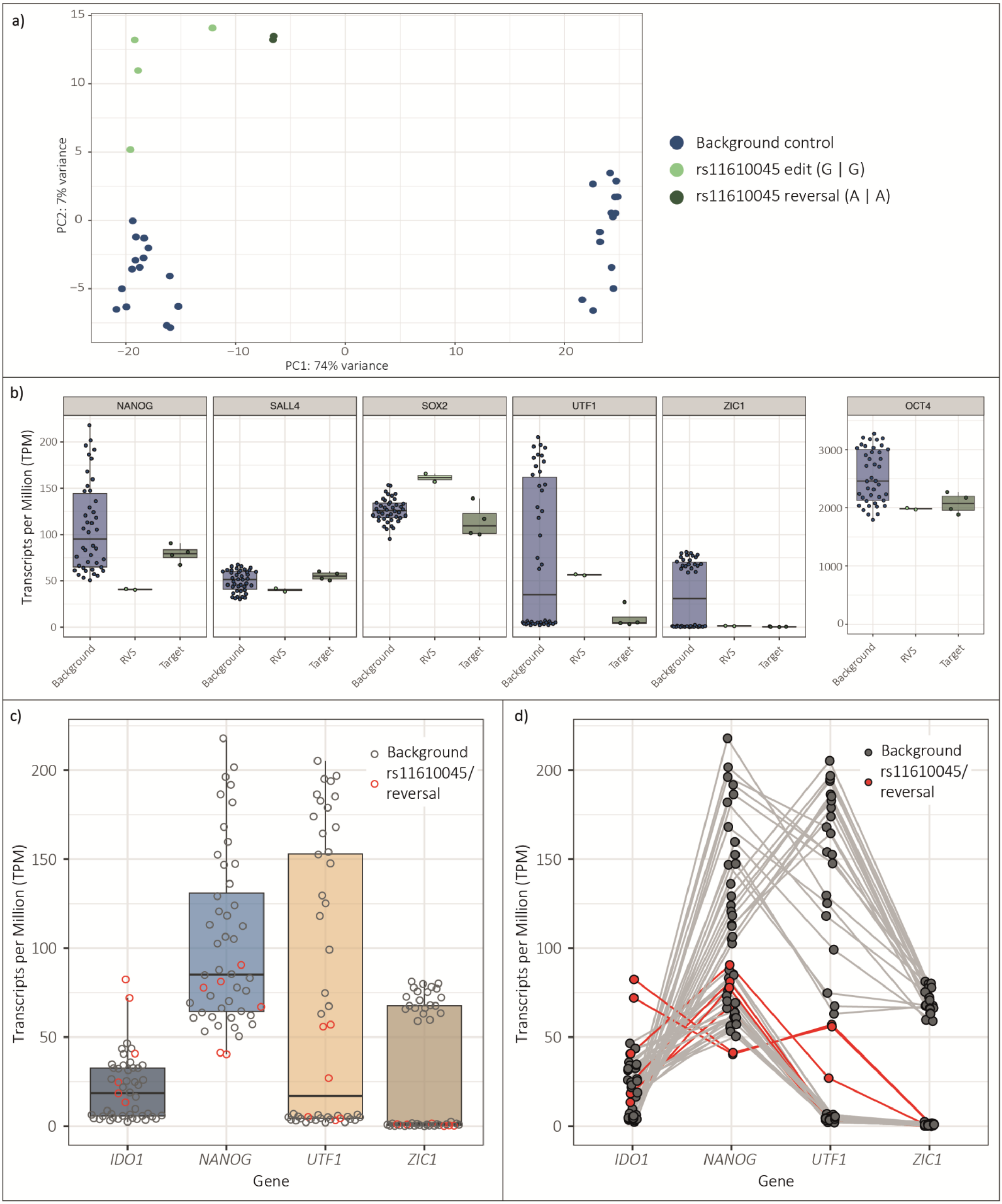

Supplementary Figure 6: iPSC cortical neuron differentiation – key marker genes. a) Gene expression levels (log10 TPM) of cortical neuron marker genes (Target – rs11610045 edited clones); b) TH gene expression levels (TPM) for background and target cell lines; c) representative immunocytochemistry images demonstrating staining for neuronal marker proteins. Antibodies used are detailed in the methods section.

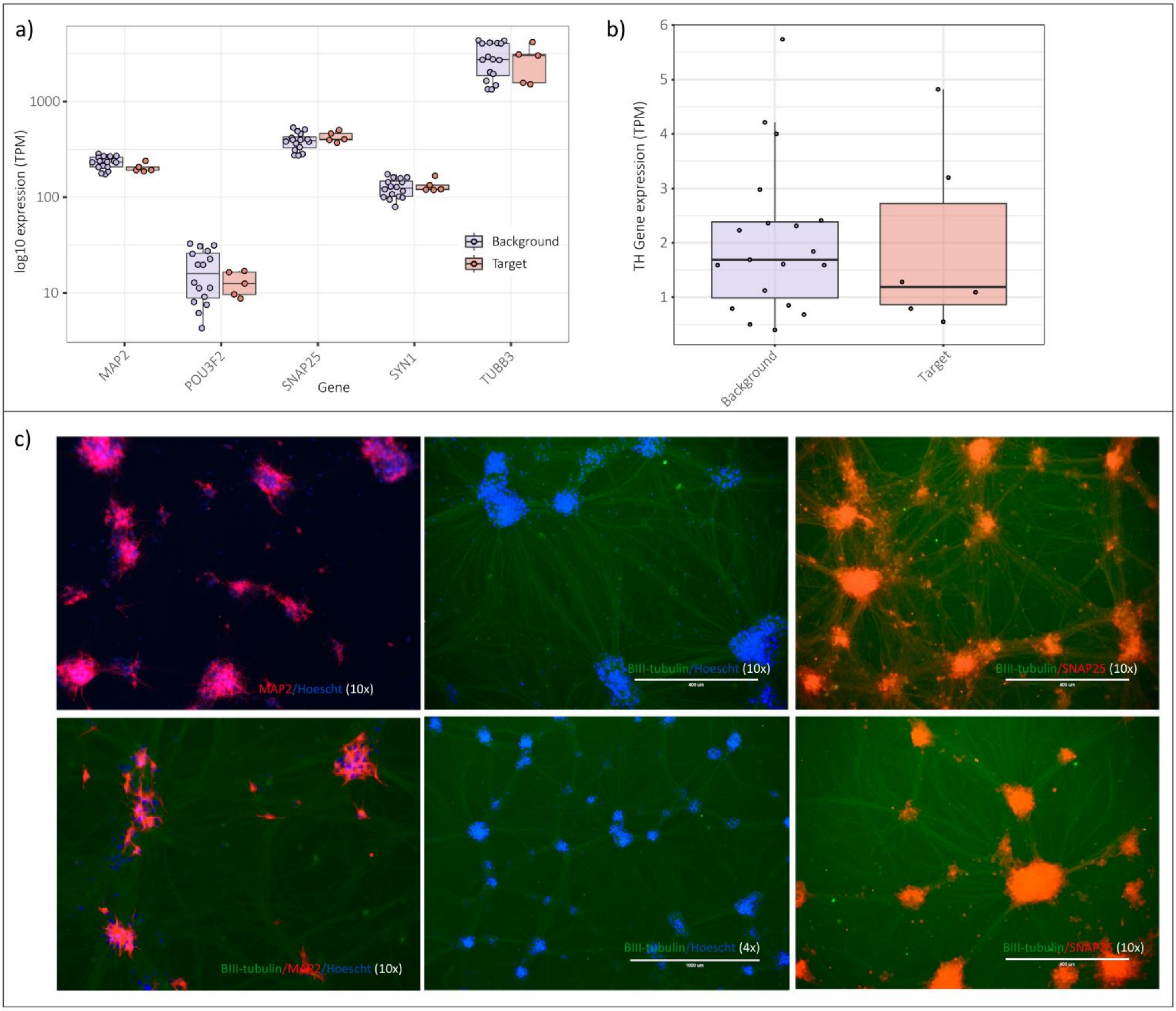

Supplement: Document S1. Figures S1–S6 [file mmc1.pdf]
